# Magnoflorine Ameliorates Lipopolysaccharide-Induced Acute Lung Injury via Suppressing NF-κB and MAPK Activation

**DOI:** 10.3389/fphar.2018.00982

**Published:** 2018-08-30

**Authors:** Shuai Guo, Kangfeng Jiang, Haichong Wu, Chao Yang, Yaping Yang, Jing Yang, Gan Zhao, Ganzhen Deng

**Affiliations:** Department of Clinical Veterinary Medicine, College of Veterinary Medicine, Huazhong Agricultural University, Wuhan, China

**Keywords:** magnoflorine, anti-inflammation, ALI, LPS, NF-κB, MAPK

## Abstract

Acute lung injury (ALI) which is featured by a strong pulmonary inflammation, is a major cause of morbidity and mortality in critically ill patients. Magnoflorine, a quaternary alkaloid isolated from Chinese herb Magnolia or Aristolochia, has been reported to have potent anti-inflammatory properties. However, the effect of magnoflorine on lipopolysaccharide (LPS)-induced ALI in mice has not been reported. The purpose of the present study is to investigate the anti-inflammatory effect of magnoflorine on LPS-induced ALI and elucidate its possible molecular mechanisms in RAW264.7 cells. The results of histopathological changes as well as the myeloperoxidase (MPO) activity indicated that magnoflorine significantly alleviated the lung injury induced by LPS. In addition, qPCR results showed that magnoflorine dose-dependently decreased the expression of pro-inflammatory cytokines TNF-α, IL-1β, and IL-6. Immunofluorescence assay also confirmed that the level of Toll-like receptor 4 (TLR4) induced by LPS was inhibited by magnoflorine treatment. Further experiments were performed using Western blotting to detect the expression of related proteins in the NF-κB and MAPK signaling pathways. The results showed that magnoflorine suppressed the levels of phosphorylated p65, IκBα, p38, ERK, and JNK. In conclusion, all data indicate that magnoflorine could protect against LPS-induced inflammation in ALI at least partially by inhibiting TLR4-mediated NF-κB and MAPK signaling pathways.

## Introduction

Acute lung injury (ALI) is a serious respiratory disease worldwide, often accompanied by symptoms of sepsis, neutrophilia, and lung inflammation (Beutz and Abraham, 2005; Matthay and Zimmerman, 2005). It is usually caused by bacteria, trauma, and pneumonia (Treggiari et al., 2004; Lim et al., 2007). Interestingly, different mechanisms are involved in the pathogenesis of ALI. Inflammation is one of the major pathogenic factors. Although the knowledge and pharmacological therapy of ALI have developed in recent decades, the mortality rate remains high (Klugman, 1990).

Lipopolysaccharide, an endotoxin released from dead Gram-negative bacteria (Wang and Quinn, 2010), could cause leukocytosis, diffuse intravascular coagulation, and endotoxic shock, which is one of the most widely used groups of stimulants in inducing ALI in mice (Takeuchi and Akira, 2010; Lu et al., 2016). TLR4 is a transmembrane protein encoded by the TLR4 gene, which is involved in the innate immune response (Takeda and Akira, 2001). There are many data indicating that LPS is the ligand of TLR4 and stimulates the inflammatory response of the lungs by binding to TLR4 (Wu et al., 2016c). It is now well established that a variety of pro-inflammatory cytokines are activated by TLR4-mediated NF-κB and MAPK signaling pathways (Xu et al., 2014; Jiang et al., 2017a). Subsequently, TNFα, IL-1β, IL-6, and other pro-inflammatory cytokines expression levels will be significantly increased (Yang et al., 2018). Therefore, blockade of TLR4-mediated NF-κB and MAPK signaling pathways can inhibit the development of ALI induced by LPS.

Magnoflorine, a quaternary alkaloid isolated from Chinese herb Magnolia (Nakano, 1954) or Aristolochia (Li and Wang, 2014), has been reported to have many biological activities, such as anti-anxiety, anti-cancer, and anti-inflammation. However, the effect of magnoflorine on LPS-induced ALI in mice has not been investigated. It has been reported that the effects of LPS on ALI can be reduced by blocking various aspects of the inflammatory cascades (Shang et al., 2010; Gong et al., 2012), indicating that magnoflorine can be used as a potential drug for the treatment of ALI. In this current research, we explored whether magnoflorine could exert its anti-inflammatory action on LPS-induced ALI in mice and in RAW264.7 cells by inhibiting the NF-κB and MAPK signaling pathways. Importantly, the results of this study can provide some reference value for the treatment of ALI in humans.

## Materials and Methods

### Reagents

**FIGURE 1:**
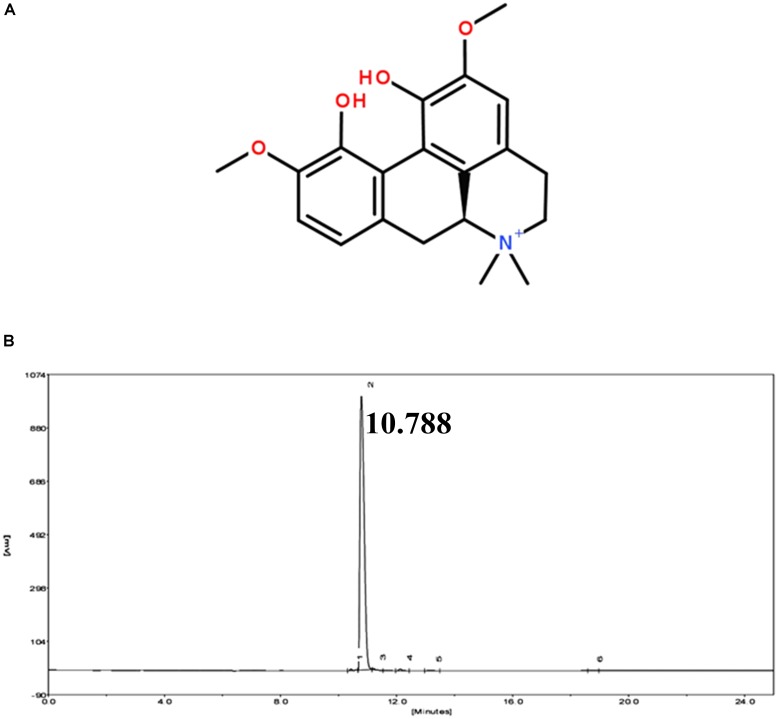
**(A)** Chemical structure of magnoflorine. **(B)** HPLC chromatogram of magnoflorine.

Magnoflorine (HPLC ≥ 98%) was obtained from Shanghai Yuanye Biotechnology Co., Ltd. (Shanghai, China) (**Figure 1**). LPS (*Escherichia coli* 055:B5) was purchased from Sigma (St. Louis, MO, United States). The myeloperoxidase (MPO) determination kits were provided by the Jiancheng Bioengineering Institute of Nanjing (Nanjing, China). The qPCR kit was obtained from Takara Bio Inc., (Otsu, Japan). NF-κB and MAPK antibodies were purchased from Cell Signaling Technology (Danvers, MA). All other chemical reagents were in accordance with the reagent specification level. All other chemical reagents meet the reagent specification standards.

### Animal Treatment and Experimental Groups

A total of 50 BALB/c male mices (6–8 weeks old, 30–35 g weight) were purchased from Wuhan Institute of Biological Products Co., Ltd. (Wuhan, China). All mices are kept in the special environment of 24°C ± 1°C, and 65% humidity, which maintain 12 h of light for 3 days to adapt to the environment before starting the experiments. During the trial, all animals were allowed to drink and feed *ad libitum*. This study was carried out in accordance with guidelines provided by the Laboratory Animal Research Center of Hubei province, and approved by the Ethical Committee on Animal Research at Huazhong Agricultural University (HZAUMO-2015-12).

The mouse were randomly divided into the following five groups of ten mice in each group for the establishment of ALI model:blank group, LPS group, Magnoflorine (5, 10, and 20 mg/kg) + LPS groups. Magnoflorine was diluted with Dulbecco’s modified Eagle’s medium (DMEM) to different concentrations. LPS was diluted with phosphate buffered saline (PBS) to a final concentration of 1 mg/ml. The method for establishing the LPS-induced ALI model was described previously (Li and Wang, 2014). Briefly, the mice were intranasally administered 50 μL of LPS to induce ALI. The blank group was intranasally administered 50 μL of PBS. After 24 h of instillation, The mice in the magnoflorine group were intraperitoneally injected with different concentrations of magnoflorine (5, 10, and 20 mg/kg) three times at 0, 8, 16 h. The blank group received equal volumes of PBS. 8 h after the last treatment with magnoflorine, the mice were were euthanized, and the lung tissue were harvested and kept at −80°C.

### High-Performance Liquid Chromatography (HPLC)

The purity of magnoflorine was measured by HPLC. The experiment was carried out using an EChrom2000 DAD data system (Elite, Dalian, China) as described previously (Wu et al., 2016d). Briefly, the separation was performed on a Hypersil ODS2-C18 analytical column (5 μm, 200 mm × 4.6 mm). Subsequently, the elution was performed using the acetonitrile-water (2:98, v/v) mobile phase. The flow rate was 1.0 mL/min, and the detection wavelength was 268 nm.

### Histopathologic Evaluation of the Lung Tissue

Lung tissues were obtained, cut into sections of approximately 0.5 cm^2^ sizes, and fixed in 10% formalin for subsequent histopathological analysis. Briefly, lung tissues were dehydrated with different concentrations of alcohol, infiltrated with xylene, embedded in paraffin, and sliced into 4 μm sections, and then stained with hematoxylin-eosin (H&E). Finally, the morphology changes of lung tissues were observed by optical microscope (Olympus).

### Myeloperoxidase Analysis

The level of MPO activity can be used to predict the early risk of inflammatory diseases (Li et al., 2015). Lung tissue was collected and ground into a tissue homogenate with a reaction buffer (w/v, 1/19), after which MPO activity was detected and analyzed according to the instructions of manufacturer’s MPO assay kit.

### Cell Viability Assay

A Cell Counting Kit-8 (CCK-8) was used for the determination of cell viability. RAW264.7 cells were grown at a density of 2 × 10^4^ cells/mL in 96 well plates. After the cells were adherent (approximately 2 h), the cells were treated with different concentrations of magnoflorine (25, 50, 100 μg/mL). After 12 h, 10 μL of CCK-8 was added in each well for 4 h at 37°C. And the OD value of the cells in each well was measured at 450 nm with a microplate reader. The cell viability = (Treatment Group OD – Blank Group OD)/(Control Group OD – Blank Group OD).

### Cell Culture and Treatment

RAW264.7 cells were purchased from the American Type Culture Collection (ATCC TIB-71^TM^). The cells were cultured in DMEM medium supplemented with 10% fetal bovine serum at 37°C with 5% CO2. The cells were pretreated with various concentrations of magnoflorine (25, 50, and 100 μg/mL) for 1 h and then stimulated with LPS (1 μg/μL) for 12 h. The cells that were not given any treatment were used as a control group.

### Quantitative PCR Assay

According to the manufacturer’s instructions, total RNA was extracted from tissues and cells using the Trizol reagent, and then cDNA was generated using a reverse transcription kit (Takara, Japan). qPCR was performed using SYBR Green plus reagent kit (Roche, Basel, Switzerland) with Light- Cycler 96 (Roche) following the instructions of the manufacturer. The expression levels of inflammatory genes were normalized to GAPDH with 2^−ΔΔC_t_^ method as described previously (Livak and Schmittgen, 2001). The primers used for qPCR are listed in **Table 1**.

**Table 1:** Primers Used for qPCR.

Name	Primer sequence (5′–3′)	GenBank accession number	Product size (bp)
TNF-α	CTTCTCATTCCTGCTTGTG ACTTGGTGGTTTGCTACG	NM_013693.3	198
IL-1β	CCTGGGCTGTCCTGATGAGAG TCCACGGGAAAGACACAGGTA	NM_008361.4	131
IL-6	GGCGGATCGGATGTTGTGAT GGACCCCAGACAATCGGTTG	NM_031168.1	199
GAPDH	CAATGTGTCCGTCGTGGATCT GTCCTCAGTGTAGCCCAAGATG	NM_001289726.1	124

### Immunofluorescence Staining

RAW264.7 cells (1 × 10^5^ cells mL^−1^) were seeded onto a six-well-plate and then were pretreated with various concentrations of magnoflorine (25, 50, and 100 μg/mL) for 1 h and then stimulated with LPS (1 μg/μL) for 12 h. The cells were fixed with 4% paraformaldehyde for 10 min, permeabilized with 0.2% Triton X-100 for 10 min, blocked with 5% BSA for 1 h and followed by incubation with rabbit anti-p-p65 antibody and anti-TLR4 antibody overnight at 4°C. Subsequently, the cells were washed and incubated with FITC-labeled goat anti-rabbit IgG antibody for 1 h. Nuclei were stained with DAPI for 10 min, and the p-p65 and TLR4 were observed using a fluorescence microscope (Olympus, Japan).

### Western Blot Analysis

Lung tissues and RAW264.7 cells were lysed with a lysate containing a phosphatase inhibitor and then centrifuged at 4°C and 12,000 rpm for 15 min. The obtained protein was measured for its concentration by a Biosharp protein measuring kit. Subsequently, sodium dodecyl sulphonate polyacrylamide gel electrophoresis was performed and 40 μg protein was loaded per well (at the same concentration). The separated protein was transferred to polyvinylidene difluoride membrane and blocked in blocking solution for 2 h, and then incubated overnight at 4°C with primary antibodies (1:1000). Afterward, the membranes were incubated with secondary antibodies (1:4000) for 1 h at 25°C. The protein levels were detected with an enhanced chemiluminescence reagent, and the intensities were quantified using Image J gel analysis software.

### Statistical Analyses

The SPSS software 16.0 (SPSS Inc.) was used for the statistical analyses. Statistical data were expressed as the mean ± SEM of three individual experiments. The data were analyzed using ANOVA followed by Dunnet’s *post hoc* test. *P*-values less than 0.05 were deemed statistically significant differences.

## Results

### Effects of Magnoflorine on LPS-Induced Lung Injury in Mice

**FIGURE 2:**
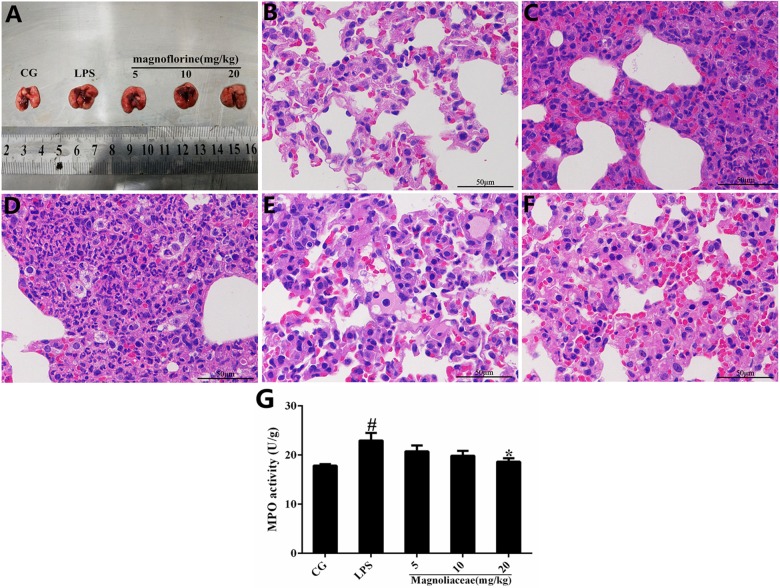
Effects of magnoflorine on LPS-induced lung injury. **(A)** Morphology of the lung. **(B)** Control group. **(C)** LPS group. **(D–F)** Magnoflorine (5, 10, and 20 mg/kg) groups. **(G)**. MPO activity assay. CG is the control group. LPS is the LPS-stimulated group. The values are presented as means ± S.E.M. of three independent experiments. ANOVA, *p* < 0.0001, *post hoc* ^#^*p* < 0.05 vs. control group, ^∗^*p* < 0.05 vs. LPS group.

Histopathological analysis and MPO assay were used to determine lung tissue damage (**Figure 2A**). There were no histopathological lesions in the control group (**Figure 2B**), whereas pathological changes such as infiltration of inflammatory cells and alveolar hyperemia were observed in the LPS group (**Figure 2C**). Interestingly, compared with the LPS group, the infiltration of inflammatory cells and the extent of alveolar congestion were significantly reduced in the magnoflorine groups (**Figures 2C–F**). A further MPO test was also used to analyze the effect of magnoflorine on LPS-induced lung injury. The results showed that LPS dramatically increased MPO activity, which was significantly reduced with magnoflorine treatment (**Figure 2G**).

### Effects of Magnoflorine on Cell Viability

**FIGURE 3:**
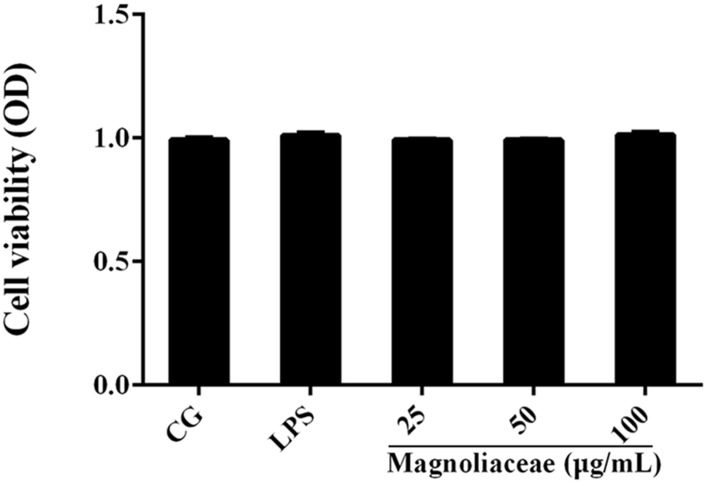
The effects of magnoflorine on cell viability. RAW 264.7 cells were cultured with LPS (1 μg/mL) and different concentrations of magnoflorine (25, 50, and 100 μg/mL) for 12 h, and then the cell viability was measured using the CCK-8 assay. The values are presented as means ± S.E.M. of three independent experiments.

The potential cytotoxicity of magnoflorine on RAW264.7 cells was determined using the CCK-8 assay. The results show that magnoflorine has no effect on cell viability (**Figure 3**).

### Effects of Magnoflorine on the Levels of Cytokines

**FIGURE 4:**
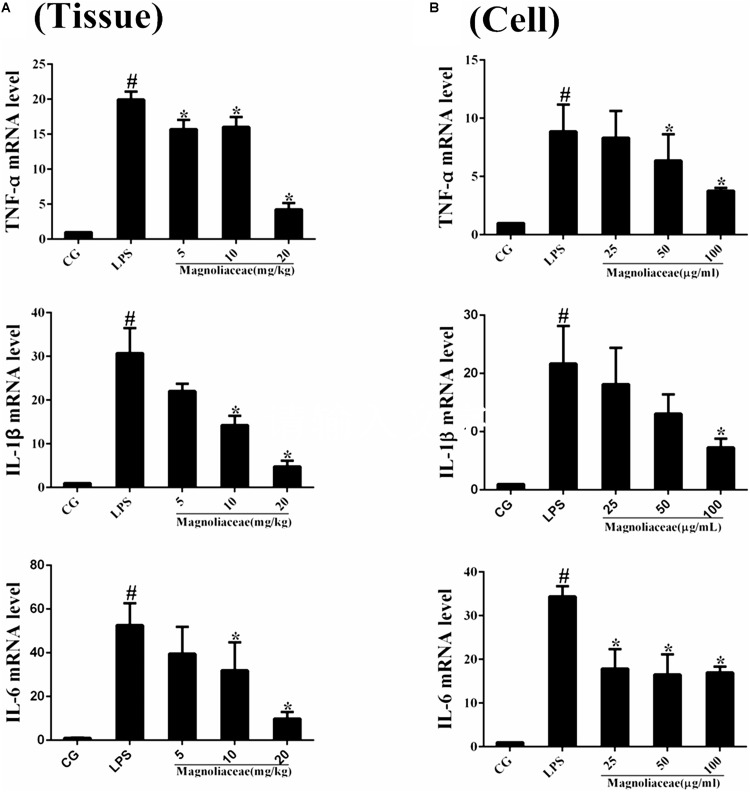
Effects of magnoflorine on the production of cytokines. **(A)** The expression of TNF-α, IL-1β, and IL-6 mRNA in lung tissues were measured by qPCR. **(B)** The expression of TNF-α, IL-1β, and IL-6 mRNA in RAW264.7cells were measured by qPCR. GAPDH was used as a control. CG is the control group. LPS is the LPS-stimulated group. The data are presented as the mean ± S.E.M. of three independent experiments. ANOVA, *p* < 0.0001, *post hoc* ^#^*p* < 0.05 vs. control group, ^∗^*p* < 0.05 vs. LPS group.

The expression levels of inflammatory cytokines in lung tissue and RAW264.7 cells were examined by qPCR. The results of the qPCR assay showed that the expression levels of TNF-α, IL-1β, and IL-6 in the LPS group were significantly higher than those in the control group. The expression levels of the three inflammatory factors in the magnoflorine group were dose-dependently reduced compared to the LPS group (**Figures 4A,B**).

### Magnoflorine Inhibition of the Expression of TLR4

**FIGURE 5:**
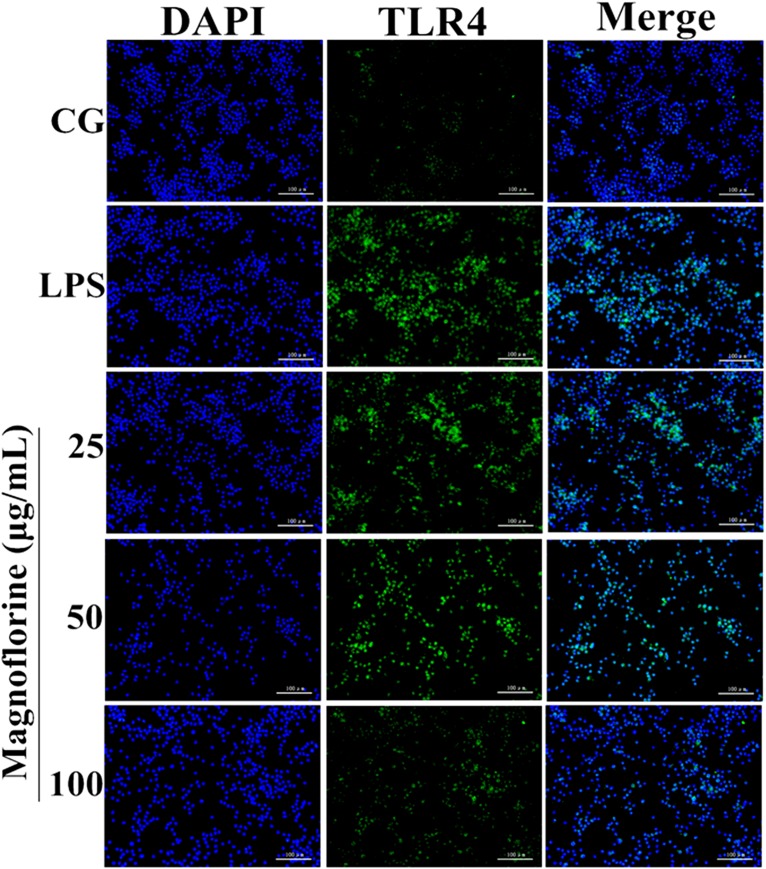
Effects of magnoflorine on the expression level of TLR4 protein. Immunofluorescence staining was performed to identify the expression of TLR4 (×200), scale bar = 100 μm. Blue spots represent cell nuclei, and green spots indicate TLR4 staining. CG is the control group. LPS is the LPS-stimulated group.

TLR4 is the first TLR receptor protein to play a role in the LPS reaction (Jiang et al., 2018), which is of great significance in LPS-induced ALI. As shown by Immunofluorescence assay, LPS group significantly increased TLR4 expression. However, the expression levels of TLR4 protein were decreased by magnoflorine groups (**Figure 5**).

### Effects of Magnoflorine on the NF-κB Pathway in LPS-Induced ALI

**FIGURE 6:**
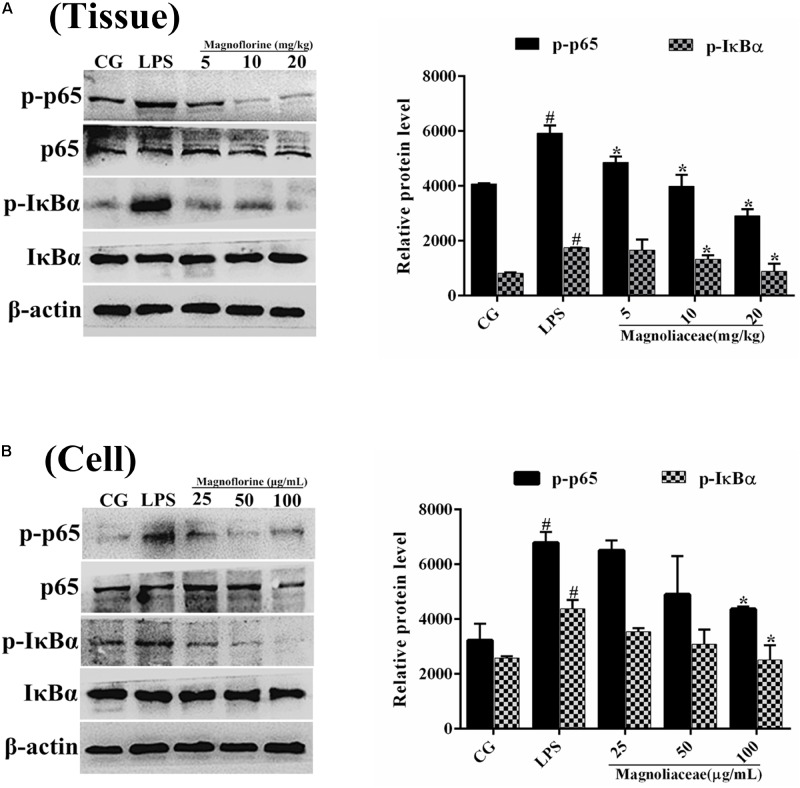
The effects of magnoflorine on the NF-κB pathway activation. **(A)** The expression levels of IκBα and p65 proteins were analyzed using specific antibodies in lung tissues. **(B)** The expression levels of IκBα and p65 proteins in RAW264.7 cells. β-actin was used as the control. CG is the control group. LPS is the LPS-stimulated group. The data represent the mean ± S.E.M. ANOVA, *p* < 0.0001, *post hoc* ^#^*p* < 0.05 vs. control group, ^∗^*p* < 0.05 vs. LPS group.

**FIGURE 7:**
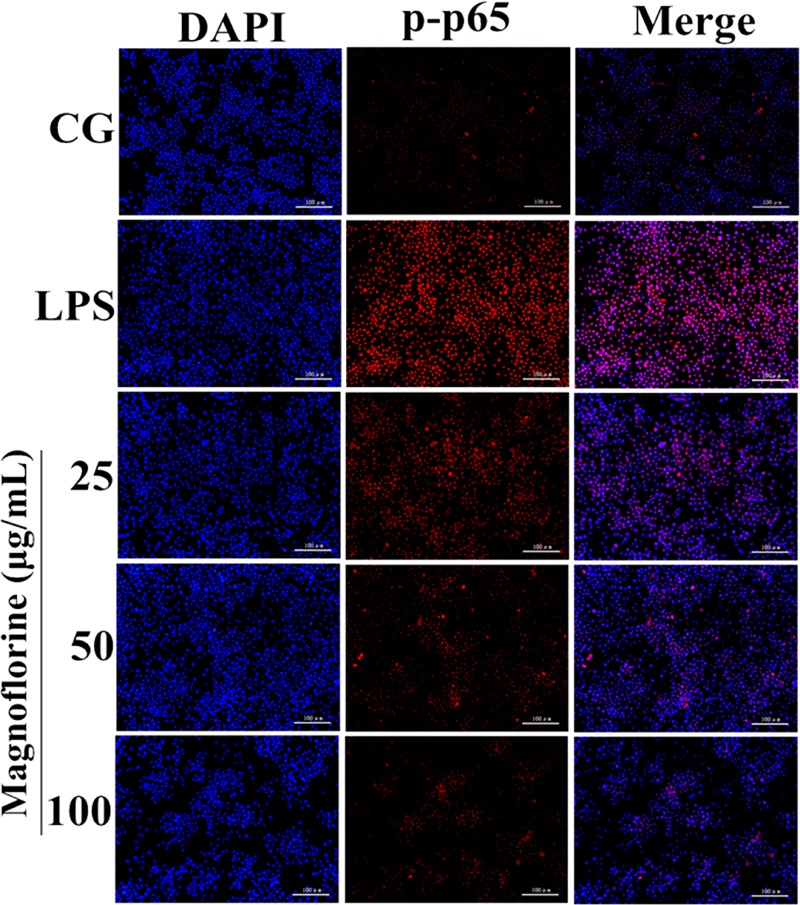
Effects of magnoflorine on p65 translocation into the nucleus. Translocation of the p65 subunit from the cytoplasm into the nucleus was assessed by immunofluorescence staining (×200), scale bar = 100 μm. Blue spots represent cell nuclei, and red spots indicate p-p65 staining. CG is the control group. LPS is the LPS-stimulated group.

NF-κB signaling pathway is one of the important signaling pathways of inflammatory response. In order to further test the effect of magnoflorine on LPS-induced NF-κB signaling pathway, the expression of NF-κB p65 and IκBα protein was detected by Western blot. The results showed that the expression of phosphorylated p65 and IκBα protein in the lung tissue was significantly higher than that in the control group. Interestingly, the expression of magnoflorine groups were relatively reduced (**Figure 6A**). Furthermore, in RAW264.7 cells, the expression levels of phosphorylated p65 and IκBα proteins were significantly higher than those in the control group, whereas the expression of the magnoflorine protein decreased in a dose-dependent manner (**Figure 6B**). To further confirm these observations, We examined the nuclear translocation of p65 protein in RAW264.7 cells. We found that the expression of nuclear p65 was significantly reduced after treatment with magnoflorine (**Figure 7**).

### Effects of Magnoflorine on the MAPK Pathway in LPS-Induced ALI

**FIGURE 8:**
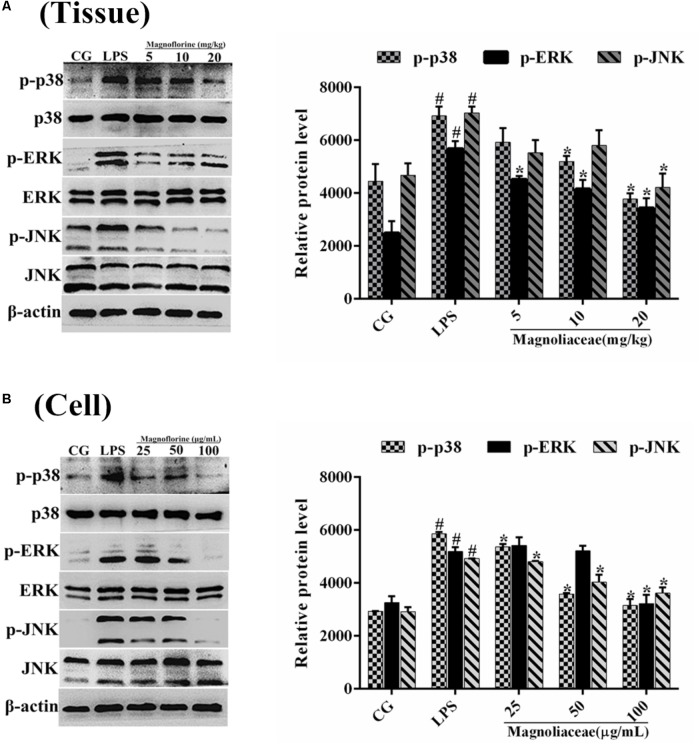
Effects of magnoflorine on the MAPK pathway activation. **(A)** The expression levels of p38, ERK, and JNK proteins in lung tissues. **(B)** The expression levels of p38, ERK, and JNK proteins in RAW264.7 cells. β-actin was used as the control. CG is the control group. LPS is the LPS-stimulated group. The data represent the mean ± S.E.M. ANOVA, *p* < 0.0001, *post hoc* ^#^*p* < 0.05 vs. control group, ^∗^*p* < 0.05 vs. LPS group.

Compared with NF-κB, MAPK is also a very important signaling pathway. The inhibitory effect of magnoflorine on the MAPK signaling pathway was evaluated by measuring the expression levels of p38, ERK and JNK proteins. The results showed that in the lung tissue, the expression of phosphorylated p38, ERK, and JNK proteins was significantly increased in the LPS group compared with the control group. In contrast, the expression levels of phosphorylated p38, ERK, and JNK proteins in the magnoflorine groups were dose-dependently lower than the LPS group (**Figure 8A**). In addition, in RAW264.7 cells, LPS phosphorylated p38, ERK, and JNK protein expression levels were significantly higher than the control group, while the expression of magnoflorine groups were relatively reduced (**Figure 8B**).

## Discussion

Although inflammation is considered as a protective mechanism elicited by the host in answer to various aggressions such as microbial infections, excessive inflammation often causes extensive tissue damage and even systemic dysfunction (Kuriakose et al., 2013; Chen et al., 2015). ALI is characterized by obvious acute inflammation with elevated pro-inflammatory cytokines levels, and is a major cause of morbidity and mortality in critically ill patients (Wu et al., 2016b). Recent studies have shown that magnoflorine has a certain anti-inflammatory effect (Li and Wang, 2014). Besides, magnoflorine has also been shown to possess potent an-tiradical and an-tioxidant activities (Rackovã et al., 2004), and this feature is typically related to the secondary metabolites with free phenolic structure such as resveratrol and apigenin (Chiavaroli et al., 2010; Menghini et al., 2016b). In addition, magnoflorine also have many biological activities, such as anti-anxiety, and anti-cancer (Li and Wang, 2014). Importantly, it can protect the oxidation of human low density lipoprotein (Hung et al., 2007). However, the effect of magnoflorine on LPS-induced ALI in mice has not been reported. In the present study, we investigated the anti-inflammatory effect of magnoflorine on LPS-induced ALI *in vivo* and *in vitro*.

It is well-known that inflammation can damage the normal lung structure and cause exudation of inflammatory products (Driver, 2012). Through the histopathological observation, we found that magnoflorine inhibited the infiltration of inflammatory cells and restrained the alveolar structural damage. Importantly, evidence has been increasing that oxidative stress could induce aberrant activation of macrophages and then results in inflammatory damage (Cachofeiro et al., 2008). Hence, the radical scavenger property of magnoflorine may be a possible mechanism of action related to the observed protective effects. It has been reported that MPO is a biomarker of neutrophil migration into tissues, which can reflect the number of neutrophils in inflamed or injured tissues (Jiang et al., 2017b). Moreover, MPO as an important therapeutic target in the treatment of inflammatory conditions and its activity reflects the infiltration of neutrophils into lung tissues (Odobasic et al., 2014). The results of the MPO assay showed that magnoflorine markedly reduced MPO activity in LPS-induced ALI, suggesting that magnoflorine could repress neutrophil influx into lung tissues. As an important immune cell, RAW 264.7 murine macrophages have been widely used in the establishment of mouse inflammation model in ALI *in vitro*. Thus, we explored the effects of magnoflorine on LPS-stimulated RAW264.7 cells. The macrophage is the important sensory and regulatory cell in immunological system; thus, we also examined the effect of magnoflorine on LPS-stimulated RAW264.7 macrophages. The CCK-8 assay showed that the different doses of magnoflorine have no toxicity to cells, consistent with a previous study.

Proinflammatory cytokines appear in the early stages of inflammation (Giebelen et al., 2007), which indicate the severity of ALI in a certain sense. LPS stimulation releases inflammatory cytokines such as TNF-α, IL-1β, IL-6, and increases their expression levels (Zhang et al., 2011). TNF-α is an important cytokine secreted by macrophages that promotes the activation of neutrophils and the release of other cytokines (Akira et al., 1990; Wu et al., 2015). Similar to TNF-α, IL-1β is also secreted by macrophages and, to some extent, regulates the progress of the inflammatory response (Akira et al., 1990). IL-6 maintains tissue homeostasis and reflects the extent of tissue damage, which is critical in the inflammatory response (Cronin et al., 2016). In addition, IL-6 could also exert a downregulating effect on pro-inflammatory TNFα (Menghini et al., 2016a). In our study, the TNF-α, IL-1β, and IL-6 levels in lung tissues and macrophages were evidently lower in the magnoflorine groups than in the LPS group. These results revealed that magnoflorine exerted anti-inflammatory effects, perhaps by reducing the levels of pro-inflammatory cytokines.

TLR4, a member of the toll-like receptor family, plays an important role in the innate immune response (Takeda and Akira, 2004; Mateu et al., 2015). Previous reports have shown that TLR4 participates in LPS-induced immune responses by activating the NF-κB and MAPK signaling pathways (Wu et al., 2016a). To further enlighten the mechanism by which magnoflorine exerts its potent anti-inflammatory action, we then explored the TLR4-mediated activation of the NF-κB and MAPK signaling pathways. We found that LPS significantly increases the expression of TLR4, while magnoflorine treatment reduced TLR4 expression to varying degrees. It has been reported that both NF-κB and MAPK signaling pathways are involved in LPS-induced mice ALI (Lin et al., 2018). NF-κB, a critical factor linking inflammation and tumorigenesis, consists of p50, p52, p65, RelB, and c-Rel, and among them p65 is one of the most studied protein (Hayden and Ghosh, 2004; Jiang et al., 2016). The activation of signaling may reflect the severity of inflammation to some extent (Liu et al., 2017). Under normal conditions, NF-κB p65 subunit and its inhibitory protein IκBα are in a resting state. Under LPS stimulation, IκBα is phosphorylated, and P65 is transferred into the nucleus and induces an inflammatory response. MAPK signaling pathway has also been reported to play an essential role in the TLR4-mediated inflammatory response (Lai et al., 2017), and can activate AP-1 and then induce the production of pro-inflammatory cytokines (Ding et al., 2010). Our results showed that magnoflorine remarkably suppressed the phosphorylation of NF-κB and MAPK *in vivo* and *in vitro*.

**FIGURE 9:**
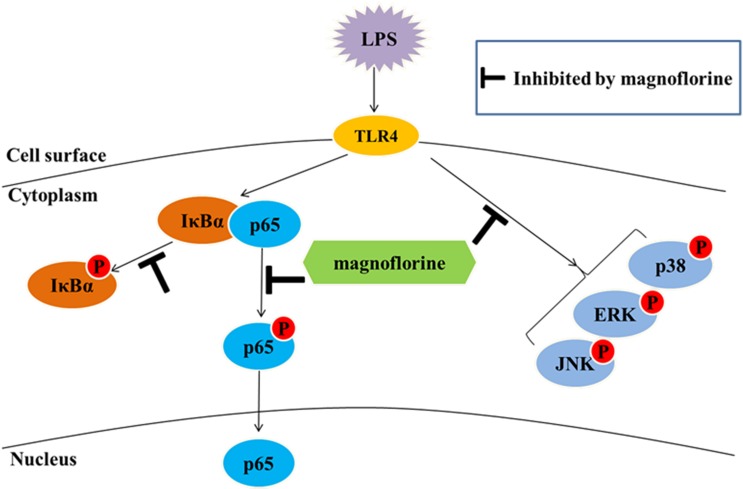
NF-κB and MAPK pathways in LPS-stimulated ALI.

In summary, our studies indicate that magnoflorine exerts its anti-inflammatory effects by reducing the expression of inflammatory factors in LPS-induced ALI. The possible mechanisms are associated with the inactivation of TLR4-mediated NF-κB and MAPK signaling pathways (**Figure 9**). Importantly, magnoflorine can pass through connection of inflammatory factors and NF-κB and MAPK signaling pathways *in vivo* and *in vitro*. Finally, it is hoped that magnoflorine might become a potential therapeutic agent for the treatment of LPS-induced ALI.

## Author Contributions

SG and KJ conceived and designed the experiments. KJ, CY, and YY carried out the experiments. JY, GZ, and HW analyzed the data. SG and GD wrote the manuscript. All authors agree to be responsible for the content of the work.

## Conflict of Interest Statement

The authors declare that the research was conducted in the absence of any commercial or financial relationships that could be construed as a potential conflict of interest. The reviewers, LB and CF, and the handling Editor declared their shared affiliation.
